# Towards Lifespan Automation for *Caenorhabditis elegans* Based on Deep Learning: Analysing Convolutional and Recurrent Neural Networks for Dead or Live Classification

**DOI:** 10.3390/s21144943

**Published:** 2021-07-20

**Authors:** Antonio García Garví, Joan Carles Puchalt, Pablo E. Layana Castro, Francisco Navarro Moya, Antonio-José Sánchez-Salmerón

**Affiliations:** Instituto de Automática e Informática Industrial, Universitat Politècnica de València, 46022 Valencia, Spain; angar25a@upv.edu.es (A.G.G.); juapucro@doctor.upv.es (J.C.P.); pablacas@doctor.upv.es (P.E.L.C.); franamo4@etsii.upv.es (F.N.M.)

**Keywords:** *C. elegans*, lifespan automation, deep learning, computer vision

## Abstract

The automation of lifespan assays with *C. elegans* in standard Petri dishes is a challenging problem because there are several problems hindering detection such as occlusions at the plate edges, dirt accumulation, and worm aggregations. Moreover, determining whether a worm is alive or dead can be complex as they barely move during the last few days of their lives. This paper proposes a method combining traditional computer vision techniques with a live/dead *C. elegans* classifier based on convolutional and recurrent neural networks from low-resolution image sequences. In addition to proposing a new method to automate lifespan, the use of data augmentation techniques is proposed to train the network in the absence of large numbers of samples. The proposed method achieved small error rates (3.54% ± 1.30% per plate) with respect to the manual curve, demonstrating its feasibility.

## 1. Introduction

In recent decades, the nematode *Caenorhabditis elegans (C. elegans)* has emerged as a biological model for the study of neurodegenerative diseases and ageing. Their size (approximately 1 mm in length) enables their cultivation and handling in standard Petri dishes in a cost-effective way, and their transparent body makes it possible to observe their organs and tissues under a microscope. The complete sequence of the *C. elegans* genome, which is similar to that of humans, has been known since 1998 [1]; moreover, its short lifespan (2 to 3 weeks) allows trials to be run in a short time period.

All these characteristics make this nematode an ideal model for the study of ageing. Among the assays performed with *C. elegans* to study ageing, one of the most outstanding is the lifespan assay [2], which consists of counting live nematodes on test plates periodically [3]. The experiment starts from the beginning of adulthood and ends when the last nematode dies. Using this count, survival curves are created, representing the survival percentage of the population each day. In this way, the different factors affecting life expectancy can be compared and contrasted.

In general, survival is determined by whether movement is observed (alive) or not (dead). However, in the last days of the experiment, *C. elegans* become stationary and only make small head or tail movements. Therefore, it is necessary to touch the body of the nematode with a platinum wire to check whether there is a response.

Today, in many laboratories, time-consuming and laborious handling and monitoring tasks are performed manually. Automation is, therefore, an attractive proposition, saving time, providing constant monitoring, and obtaining more accurate measurements.

Automatic monitoring of *C. elegans* cultured in standard Petri dishes is a complex task due to (1) the great variety of forms or poses these nematodes can adopt, (2) the problem of dirt and condensation on the plate, which requires the use of special methods, and (3) the problem of aggregation of several *C. elegans*, which requires specific detection techniques.

Furthermore, discriminating between dead and live worms presents difficulties as they hardly move in the last few days of their lives, requiring greater precision, longer monitoring times to confirm death and, therefore, higher computational and memory costs.

In the literature [4], major contributions can be found that have proposed solutions to the problem of automating lifespan experiments with *C. elegans*. Some outstanding examples are given below. WormScan [5] was one of the first works to use scanners to monitor *C. elegans* experiments and determine whether a nematode is alive or dead on the basis of its movement; Lifespan Machine [6] also monitors petri dishes with scanners but with improved optics and systems to control the heat generated by the scanners. In addition, the authors developed their own software to determine whether nematodes are alive or dead. To classify dead worms, they identified stationary worms and track small posture changes to determine the time of death. WorMotel [7] uses specific plates with multiple microfabricated wells, thus allowing individual nematodes to be analysed and avoiding the problem of aggregation. Automated Wormscan [8] takes the WormScan method and makes it fully automatic. WormBot [9] is a robotic system that allows semi-automatic lifespan analysis. Lastly, a method based on vibration to stimulate *C. elegans* in Petri plates, to confirm whether worms are dead or alive, was proposed in [10].

These methods use traditional computer vision techniques that require feature design and manual adjustment of numerous parameters. In recent years, the rise of deep learning has led to breakthroughs in computer vision tasks such as object detection, classification, and segmentation [11,12,13]. There has been a shift from a feature design paradigm to one of automatic feature extraction.

To date, no studies have reported automated lifespan assays with *C. elegans* using artificial neural networks. However, there has been an increase in the number of studies using machine learning and deep learning to solve other problems related to these nematodes. For example, a *C. elegans* trajectory generator using a long short-term memory (LSTM) was proposed in [14]. WorMachine [15] is a tool that uses machine learning techniques for the identification, sex classification, and extraction of different phenotypes of *C. elegans*. A support vector machine (SVM) was used for the automatic detection of *C. elegans* via a smartphone app in [16]. A method that classifies different strains of *C. elegans* using convolutional neural networks (CNN) was presented in [17]. Methods based on neural networks have also been proposed for head and tail localisation [18] and pose estimation [19,20,21]. Recently, [22,23] used different convolutional neural network models to estimate the physiological age of *C. elegans*. A method for the identification and detection of *C. elegans* based on Faster R-CNN was proposed in [24]. Lastly, [25] developed a CNN that classifies young adult worms into short-lived and long-lived. They also used this CNN to classify worm movement.

This article proposes a method using simple computer vision techniques and neural networks to automate lifespan assays. Specifically, it is a classifier that determines whether a *C. elegans* is alive or dead by analysing a sequence of images. The architecture combines a pretrained convolutional neural network (Resnet18) with a recurrent LSTM network. In addition to proposing a method to automate lifespan, the use of data augmentation techniques (mainly based on a simulator) has been proposed to train the network despite the lack of a large number of samples. Our method obtained 91% accuracy in classifying *C. elegans* image sequences as alive or dead from our validation dataset.

After training and validating the classifier, this method was tested by automatically counting *C. elegans* on several real lifespan plates, slightly improving the results of an automatic method based purely on traditional computer vision techniques.

The article is structured as follows: the proposed method is presented in Section 2, the experiments are reported in Section 3, and the results are discussed in Section 4.

## 2. Materials and Methods

### 2.1. C. elegans Strains and Culture Conditions (Lifespan Assay Protocol)

The *Caenorhabditis* Genetics Centre at the University of Minnesota provided the *C. elegans* of the strains N2, Bristol (wild-type), and CB1370, *daf-2 (e1370)* that were used to perform the lifespan assays.

All worms were age-synchronised and pipetted onto Nematode Growth Medium (NGM) in 55 mm Petri plates. Temperature was maintained at 20 °C. In order to reduce the probability of reproduction, FUdR (0.2 mM) was added to the plates.

Fungizone was added to reduce fungal contamination [26]. As a standard diet, strain OP50 of *Escherichia coli* was used, which was seeded in the middle of the plate as worms tend to stay on the lawn, thus avoiding occluded wall zones.

The procedure followed by the laboratory operator on every day of the assay was as follows: (1) He removed the plates from the incubator and placed them in the acquisition system; (2) before starting the capture, he made sure that there was no condensation on the lid and removed it if detected; (3) he captured a sequence of 30 images per plate at 1 fps and returned the plates to the incubator. This reduced the time that the plates were out of the incubator prevented condensation on the lid. In addition, the room temperature was maintained at 20 °C to prevent condensation.

### 2.2. Automated Lifespan Algorithm Based on Traditional Computer Vision Techniques

To develop the new method proposed in this article, we took as a starting point the automatic lifespan method based on traditional computer vision techniques proposed in [27]. Parts of this method were taken, such as segmentation, motion detection in the edge zone of the plate, and postprocessing. In addition, this method was used as a baseline to compare the accuracy of the new method in obtaining the lifespan curves.

### 2.3. Proposed Automatic Lifespan Method

The problem of counting the number of live *C. elegans* within a plate, applying deep learning directly from plate image sequences, is a very interesting regression problem. However, neural networks require a dataset with a lot of data to feed the learning process of its millions of parameters. Considering the high cost of obtaining a labelled dataset from these image sequences, traditional computer vision techniques are proposed to simplify the problem to be solved by the neural network to a classification problem.

The proposed method solves the problem of classifying whether a *C. elegans* is alive or dead from a sequence of *C. elegans* images. This approach requires processing the image sequences from the plate, using traditional computer vision techniques to extract the image sequence of each *C. elegans*, which is the input to the classifier. Moreover, a cascade classifier is proposed. Initially, trivial cases of live *C. elegans* are detected using traditional motion detection techniques, leaving the solution of the classification problem where live *C. elegans* sequences look more similar to dead sequences as a final step for the neural network.

**Figure 1:**
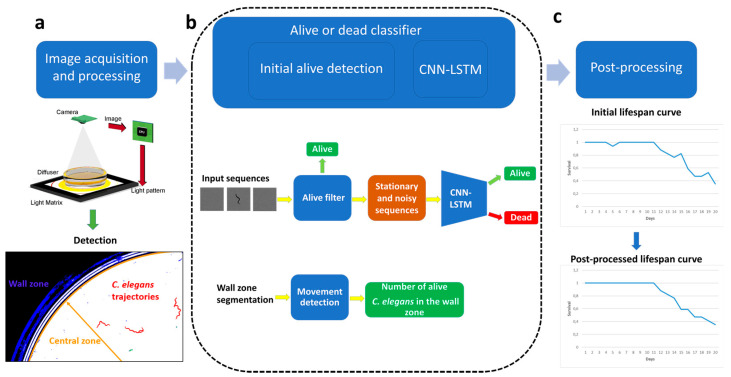
General overview of the proposed method: (**a**) capture and processing; (**b**) classification; (**c**) obtaining lifespan curve and postprocessing.

Figure 1 shows the stages of the proposed lifespan automation method. Firstly (Figure 1a), the image sequence is captured using the intelligent light system proposed in [28]. Secondly, the image sequences are processed to obtain the images for the classifier input. Then, the classifier (Figure 1b), which consists of two stages (initial detection of live *C. elegans* and alive/dead classification using a neural network) obtains the number of live and dead *C. elegans*. Then, a post-processing filter is applied to this result as described in [27], in order to correct the counting errors that may occur due to different factors (occlusions, segmentation errors due to the presence of opaque particles in the medium or the plate lid, decomposition) and, thus, finally obtain the lifespan curve (Figure 1c).

### 2.4. Image Acquisition Method

Images were captured using the monitoring system developed in [29]. This system uses the active backlight illumination method proposed in [28], which consists of placing an RGB Raspberry Pi camera v1.3 (OmniVision OV5647, which has a resolution of 2592 × 1944 pixels, a pixel size of 1.4 × 1.4 μm, a view field of 53.50° × 41.41°, optical size of 1/4′′, and focal ratio of 2.9) in front of the lighting system (a 7′′ Raspberry Pi display 800 × 480 at a resolution at 60 fps, 24 bit RGB colour) and the inspected plate in between. A Raspberry Pi 3 was used as a processor to control lighting. The distance between the camera and the Petri plate was sufficient to enable a complete picture of the Petri plate, and the camera lens was focused at this distance (about 77 mm).

With this image capture and resolution setting (1944 × 1944 pixels), the worm size projects approximately 55 × 3 pixels. Although working under these resolution conditions makes the problem more difficult, it has advantages in terms of computational time and memory.

### 2.5. Processing

The images captured by the system used have two clearly differentiated zones; on the one hand, there is the central zone, which has homogeneous illumination, and, on the other hand, there is the wall zone, which has dark areas and noisy pixels. For this reason, these areas are processed independently using the techniques described in [27].

**Figure 2:**
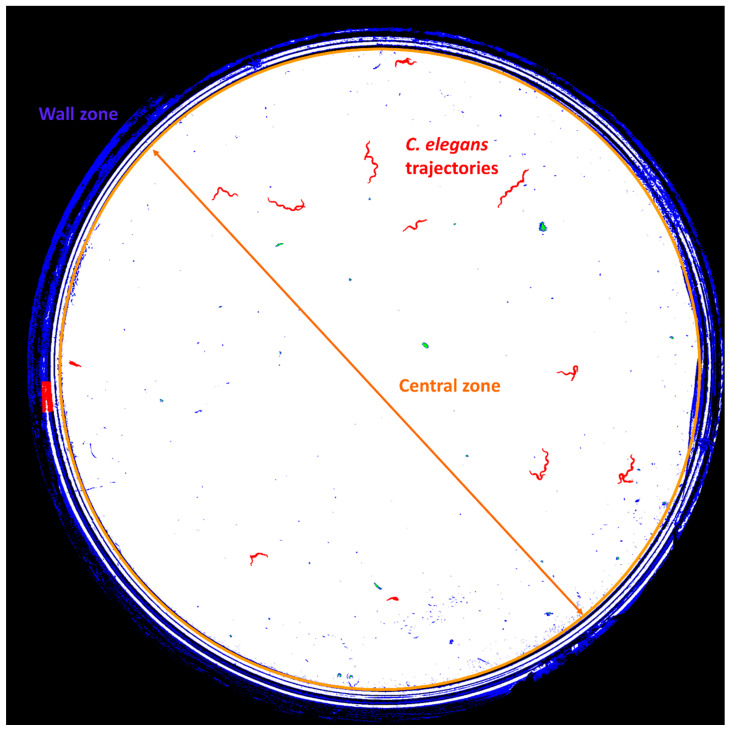
Result of the processing of the captured sequence. The orange circle delimits the central zone and the wall zone. In red are the trajectories of *C. elegans*.

The central zone (white circle delimited by the orange circumference in Figure 2) was processed at the worm tracking level (segmentation in red, Figure 2), finally obtaining the centroids of the *C. elegans* in the last of the 30 images making up the daily sequence.

In the wall zone, this tracking is impossible due to the presence of dark areas; however, the capture system generates well-illuminated white rings that allow the characteristic movements of *C. elegans* to be partially detected.

Our alive/dead criterion considers a worm to be dead when it remains in the same position and posture for more than 1 day. One way to analyse whether a nematode is alive is to compare the image of the current day with the image of the day before and the day after. Thus, by simply analysing a sequence of three images, it is possible to determine whether the worm is alive or dead.

**Figure 3:**
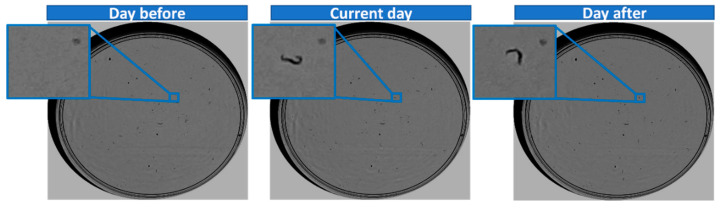
Generation of an input sequence.

To generate the image sequence in order to determine whether the worm is alive or dead, three square sub-images centred on the centroid of the current day’s *C. elegans* were cropped from the current day’s image, as well as from the previous and following days’ images, as shown in Figure 3.

The size of the sub-images was chosen taking into account that the maximum length of a *C. elegans* is approximately 55 pixels. In addition, the small displacements and rotations of the plate that occur when lab technicians place it in the acquisition system each day were also taken into account. These displacements are limited because the capture device has a system for fixing the plates. Measurements were taken experimentally to estimate the possible small displacements, obtaining a maximum displacement of 15 pixels.

The problem of plate displacements can be addressed using traditional techniques; however, achieving an alignment that works for all sequences is complicated due to potential variability of noise, illumination changes, and aggregation. For this reason, we decided not to perform alignments, but to increase the sub-image size to ensure that it appears completely within the three sub-images if the *C. elegans* is stationary.

Therefore, taking into account the maximum worm length, the maximum estimated displacement of the plate, and a safety margin of 10 pixels, the final size of the sub-images was 80 × 80 pixels, as shown in Figure 3.

### 2.6. Classification Method

From these input sequences, various approaches using traditional computer vision techniques can be considered to determine whether a *C. elegans* is alive or dead. These traditional methods require image alignment and feature design to identify the worm in cases of aggregation or fusion with opaque particles (for example, dust spots on lids) that cause segmentation errors. In addition, *C. elegans* perform small head or tail movements in the last few days, which are difficult to detect.

Our approach was based on using a two-stage cascade classifier. The aim of this cascade processing was to first classify the sequences that are clearly from live *C. elegans* and let the network decide which cases cannot be determined by the simple motion detection rules.

In the first stage, information from the wall zone was processed, and live *C. elegans* in this zone were estimated using simple motion detection methods. Conversely, in the central zone, sequences of live worms in which *C. elegans* did not appear in any of the images or moved substantially were detected using simple rules.

The remaining more complex cases (stationary *C. elegans* or with little displacement; images with noise (opaque particles causing segmentation errors)), which would require more advanced techniques and were difficult to adjust, went on to the second stage, which used a neural network to classify these cases as alive or dead.

### 2.7. Initial Detection of Live Worms

At this stage, the inputs of the different plate areas (centre and wall) were processed separately.

The wall zone was processed using the motion detection algorithm described in [27]. The irregular lighting conditions in this area made it necessary to apply movement detection techniques based on temporal analysis of changes in the segmented images. A movement was considered to correspond to *C. elegans* if the intensity changes of the pixels occurred with low frequency and exceeded an area threshold.

For the central zone, the initial detection algorithm analysed each sequence and, in each of the frames, found all the blobs that met a chosen minimum area, taking into account the minimum area that a *C. elegans* can have (area 20 pixels).

In the images for the current day, *C. elegans* was always found centred in the sub-image (except when there were segmentation errors); thus, it was easy to identify the blob and obtain its centroid. In the remaining frames, the system detected the blobs whose centroid was at a distance from the centroid of the blob of the central frame (current day) of less than a threshold of 20 pixels. This threshold was chosen taking into account the plate displacements (estimated at 15 pixels and with a safety margin of 5).

**Figure 4:**
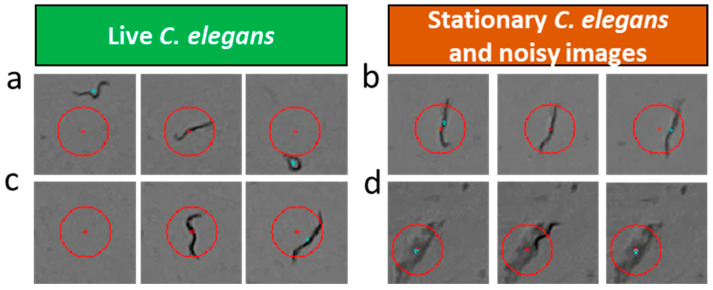
Examples of image sequences that can be classified with the initial detection method (**a**,**c**) and of the stationary (**b**) and noisy (**d**) images classified by the neural network. The red circle is centred on the centroid of the *C. elegans*/blob of the central frame and has a radius of 20 pixels. The blue dot is the centroid of the blobs detected in the remaining frames.

After obtaining the centroids of the blobs (if any) fulfilling the distance constraint, a first classification (live sequences or sequences to be processed by the neural network) was performed, taking into account the following logic: (1) if, in frame 1 (previous day) or in frame 3 (later day), no blob was found meeting the minimum area and distance to the centre constraints, it signified that the *C. elegans* moved more than the maximum distance (Figure 4a) or was not in the image (Figure 4c) and, therefore, it could be assured that it corresponded to a live worm; (2) otherwise, it may not have moved in any of the three images or it may have made small displacements (Figure 4b) or it may have fused with noise, producing a segmentation error (Figure 4d) causing non-*C. elegans* blobs to be detected.

With this simple processing method, the first stage classified the live *C. elegans* in the wall zone and the live *C. elegans* in the central zone, being easily detectable following simple rules. The remaining stationary *C. elegans* and images with noise, which were more complex to classify using traditional techniques, went on to the next classification stage with a neural network.

### 2.8. Alive/Dead Classification with the Neural Network

One of the most common approaches to designing neural network architectures for image sequence analysis is to combine convolutional neural networks with recurrent networks [30]. In this case, the convolutional network does the feature extraction and the recurrent network processes the temporal dynamics.

Based on this technique, we decided to employ an architecture using a pretrained convolutional network (Resnet18) as feature extractor, combined with a recurrent network (LSTM) and a fully connected layer to perform the classification.

**Figure 5:**
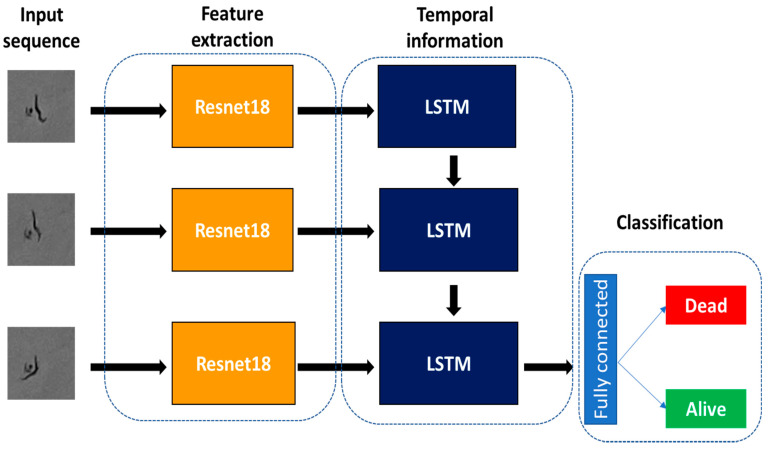
Diagram of the CNN–LSTM architecture used. The CNN performs the feature extraction, the LSTM extracts the temporal information, and the fully connected layer performs the alive or dead classification.

The Pytorch implementation of the Resnet18 [31] was used as a pretrained convolutional network, by removing the last fully connected layer. Thus, at the output of the convolutional network, a feature vector of size 512 was obtained for each input channel to the network. For initialisation, we started from the pretrained weights in the Imagenet dataset, which contained 1.2 million images of 1000 classes. Nevertheless, these weights were not fixed, but the network was completely retrained. The unidirectional LSTM network employed had a single layer and a hidden size of 256. Lastly, the features extracted by the LSTM were passed to a fully connected layer for classification. A schematic representation of the architecture used is shown in Figure 5 and details of the different layers are given in Table 1.

We created a repository on github: https://github.com/AntonioGarciaGarvi/C.-elegans-alive-dead-classification-using-deep-learning (accessed on 19 July 2021) with a demo to show some examples of how our model classifies a *C. elegans* as alive or dead using a sequence of three images corresponding to the current day, the day before, and the day after.

### 2.9. Dataset

The original dataset was obtained from images captured from 108 real assay plates containing 10–15 nematodes each using the acquisition method described.

**Figure 6:**
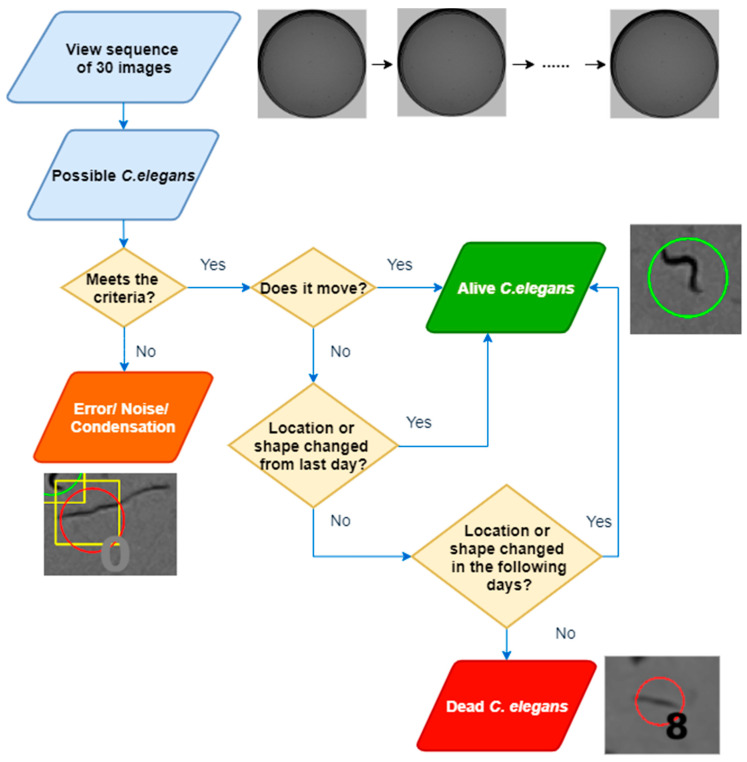
Flow chart of the labelling process.

To carry out labelling (Figure 6), the sequence of 30 images was first visualised, and possible *C. elegans* were identified. Depending on whether they met the nematode characteristics (colour, length, width, and sinusoidal movement), they were analysed in detail. If the *C. elegans* moved during the 30-image sequence, it was labelled as alive. If not, it was checked whether it was in the same position and posture on the previous and subsequent days. If no variation was observed, it was labelled as dead; otherwise, it was labelled as alive. As can be seen, this procedure is very laborious; hence, the cost of generating a labelled dataset is high.

The total number of labelled image sequences of each class is as shown in Table 2.

This dataset, as demonstrated in the experiments and results section, was insufficient for the neural network to learn to solve the proposed classification task. To solve this drawback, different types of synthetic images were generated to increase the size of the dataset. This work experimentally demonstrated that this increase in data helped to improve the results.

**Figure 7:**
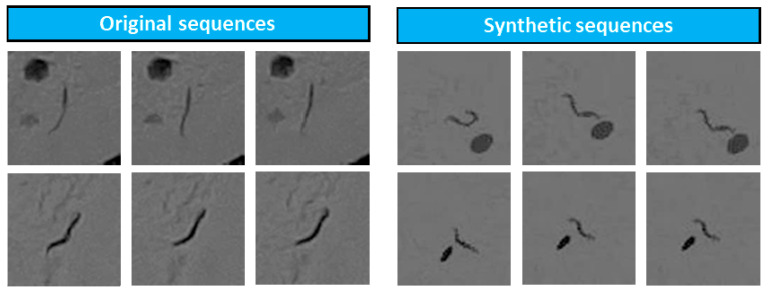
Two original image sequences are shown on the left and two sequences generated by the simulator are shown on the right.

The first types of synthetic sequences were generated with a *C. elegans* trajectory simulator (Figure 7). This simulator is based on the following components: (a) set of real images of empty Petri dishes; (b) real *C. elegans* trajectories obtained with a tracker stored in xml files; (c) colour and width features obtained from real images; (d) random positioning algorithm of the trajectories within the dish; (e) static noise generator similar to the one appearing in the original sequences.

As parameters, the simulator received the number of sequences to be generated, the number of *C. elegans* per plate, and the speed of the movement (variation between poses). To make the network learn to detect small differences between poses, the sequences that were generated had small pose changes between the previous day’s pose and the current pose, whereas the subsequent day’s was is the same as the current day’s pose. In addition, static blobs were added to these images, which also helped the network to distinguish *C. elegans* from other dark blobs which may appear in the image. Lastly, small rotations and translations were applied to the images to simulate the displacements occurring when the real plates were placed in the acquisition system. This simulator allowed us to obtain the number of sequences shown in Table 3.

As shown in Table 2, the number of dead *C. elegans* sequences was significantly lower than the number of live *C. elegans*. This is because there could only be one sequence for each dead *C. elegans*, whereas, for the live sequence, there was the whole lifespan. To train the classifier, the number of samples in each class must be balanced; therefore, a large part of the alive *C. elegans* sequences could not be used to train the network.

In order to take advantage of these remaining sequences, a second type of synthetic image was designed. These consisted of replicating the image of a *C. elegans*, thus obtaining a sequence of three images in which there was no movement or change in posture (dead *C. elegans* sequence). Small translations and rotations were applied between frames to simulate plate placement shifts.

### 2.10. Neural Network Training Method

The network was implemented and trained using the Pytorch deep learning framework [32] on a computer with an Intel^®^ Core™ i7-7700K processor and NVidia GeForce GTX 1070 Ti graphics card. The network was trained for 130 epochs using the cross-entropy loss cost function and Adam’s optimiser [33] with a learning rate of 0.0001 for 120 epochs and 0.00001 for the last 10 epochs. The batch size chosen was 64 samples taking into account memory constraints. As a regularisation and data augmentation technique, rotations (90°, 180°, and 270°) were used. The original images were resized to 224 × 224 pixels using bilinear interpolation to adapt them to the resnet input.

### 2.11. Postprocessing

As discussed above, there are different situations (occlusions, dirt, decomposition, reproduction, and aggregation) that can lead to errors in the daily live count of the lifespan curve. To alleviate these problems, the postprocessing algorithm proposed in [27] was employed. This algorithm is based on the premise that lifespan curves must be monotonically decreasing functions and, therefore, errors can be detected if the current day’s count is higher than the previous day’s count.

This correction takes into account that in the first days the errors are most likely to be false negatives due to worm occlusions at the edge and aggregations, whereas, in the last days, the errors are mostly likely due to false positives caused by plate dirt.

In this way, the lifespan cycle was divided into two periods. This division was made on the basis of the mean life, which was usually 14 days for the N2 strain. In the first cycle, the curves were corrected upwards, i.e., if the current day’s count was higher than the previous day’s count, the previous day’s count was changed to the current value. In the second cycle, they were corrected downwards, i.e., the current day’s count was decreased to the previous day’s value if it was lower.

### 2.12. Validation Method

To evaluate the proposed method, the available dataset was classified using the following validation metrics:

- The confusion matrix (Table 4), showing the number of correct and wrong predictions during model validation for each of the classes.

TD represents the actual (true) dead worms, FA represents the false live worms, FD represents the false dead worms, and TA represents the actual (true) live worms.

- The hit ratio (Equations (1) and (2)), which measure the percentage of correct predictions for a class out of all samples in that class.

(1) $$\mathrm{True}\;\mathrm{dead}\;\mathrm{rate}=\frac{\mathrm{TD}}{\mathrm{TD}+\mathrm{FA}}$$

(2) $$\mathrm{True}\;\mathrm{live}\;\mathrm{rate}=\frac{\mathrm{TA}}{\mathrm{TA}+\mathrm{FD}}$$

After evaluating the classification method, the error in the lifespan curves was tested by calculating the error between the percentage survival of the manual count and the automatic count. In addition, the results obtained were compared with those of the traditional automatic computer vision algorithm used as a reference.

The percentage of live *C. elegans* on each day of the experiment was calculated using Equation (3). The error in one day (e (d)) was the difference in absolute value between the percentage of live *C. elegans* from the manual curve and the curve from the automatic method (Equation (4)). The error per plate was the average of the errors over the days of the experiment (Equation (5)).

(3) $$\%\;\mathrm{live}\;C.\;\mathrm{elegans}=\frac{\mathrm{live}\;C.\;\mathrm{elegans}\;\mathrm{current}\;\mathrm{day}}{\mathrm{initial}\;\mathrm{live}\;C.\;\mathrm{elegans}}\times 100$$

(4) $$e\left(d\right)=\left|{\%\;\mathrm{live}\_\mathrm{manual}}_{\left(d\right)}{\;-\;\%\;\mathrm{live}\_\mathrm{automatic}}_{\left(d\right)}\right|$$

(5) $$\mathrm{ep}=\frac{\sum_{d=1}^{\mathrm{days}}e\;\left(d\right)}{\mathrm{days}}$$

The error per condition was calculated analogously, by adding up the count of all the plates for that condition, calculating the survival rates, and averaging the absolute value of the errors for each day.

## 3. Results

### 3.1. Initial Classification of the Original Dataset

First, the sequences of the original dataset (Table 2) were processed with the initial detection algorithm (first stage of the classifier).

As Table 5 shows, 81.18% of the live *C. elegans* image sequences were classified by the initial detection algorithm as a trivial live case. The remaining 18.82% were determined by the initial detection algorithm to be *C. elegans* sequences to be classified by the neural network. In the case of dead *C. elegans*, 1.65% of cases were misclassified.

The sequences that were not classified as alive by the first stage of the classifier were those used to build the training and validation dataset (Table 6) of the neural network.

### 3.2. Analysis of the NN Classifier Using Different Datasets

In this experiment, the described network was trained using (a) an original training dataset taken from the original sequences that were not classified as alive in the previous stage (1072 live sequences and 833 dead sequences), (b) a dataset generated with the simulator (Table 3), and (c) a mixed dataset, consisting of the original images, those generated by the simulator, and those generated by replicating images of live *C. elegans*. However, all models were evaluated using the original validation dataset.

Since the number of sequences from each class had to be balanced, 833 sequences from each class could be used, leaving 239 live sequences unused.

To build the original training and validation dataset (Table 6), it was considered that at least a validation sample of 1000 sequences, i.e., 500 of each class, should be available. Therefore, 333 images of each class remained to train the neural network.

The mixed dataset (Table 7) was formed using the images from the simulated dataset, images from the original dataset, the original live images that were not used in order to keep the dataset balanced (239), and the same number of dead images generated from the live images.

The results obtained (Table 8) when evaluating the different models with the original validation dataset showed how using synthetic data improved the results by 10.40% and how mixing original and synthetic data further increased the hit rates.

Table 9 shows the confusion matrix obtained by validating the model trained with the mixed dataset with the original dataset.

### 3.3. Overall Accuracy Analysis of the Cascade Classifier

The confusion matrix of all *C. elegans* sequences classified by the proposed method, i.e., live *C. elegans* classified at the initial detection stage (4624), and live and dead *C. elegans* from the validation dataset classified by the network, is shown below (Table 10). The hit ratio is shown in Table 11.

### 3.4. Validation of the Lifespan Method

In order to properly validate the automatic lifespan method, it was not enough to analyse the hit ratio of the classifier; indeed, it was necessary to measure the final error on the lifespan curves.

For this purpose, a lifespan assay was performed with eight plates following the culture and acquisition procedures described in this article. These plates were independent of the data used in the training and validation of the classification method. Each plate had between nine and 20 *C. elegans* of the N2 strain, for a total of *n* = 111.

The manual count performed following the labelling method described above was taken as ground truth. In addition to comparing the results of the proposed method with the manual results, it was compared with the automatic method proposed in [27] to obtain a comparison with another lifespan automation method.

**Figure 8:**
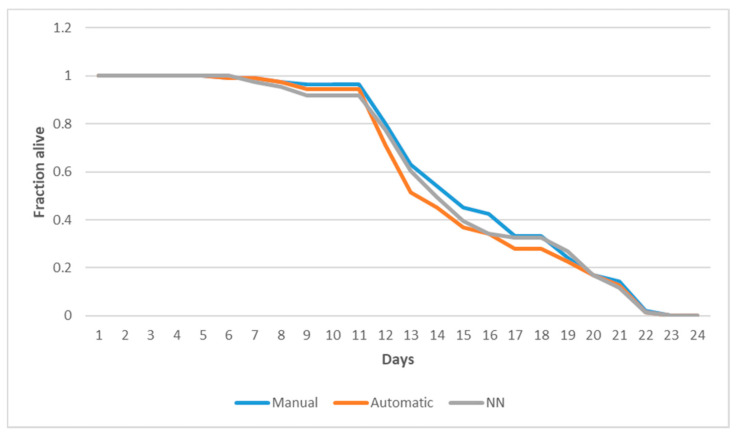
Comparison of manual counting (blue curve), automatic counting traditional method (orange curve), and counting with the proposed method (grey curve). The horizontal axis shows the days of the experiment, and the vertical axis shows the proportion of live *C. elegans*. Capture was performed until day 21, and the remaining days were approximated using a Kaplan–Meier estimator.

The curve obtained with the proposed method showed an average error in the analysis per plate of 3.54% ± 1.30%, while the traditional algorithm obtained 3.82% ± 1.76%. In the analysis of the error per condition (Figure 8), an average error was obtained on each day of 2.30% ± 2.31%, compared to 3.20% ± 3.96% for the traditional algorithm.

A statistical significance analysis was carried out with the aim of (1) analysing the differences between the manual curve and the curve obtained with the new method based on neural networks, and (2) analysing whether the improvements obtained with the new method with respect to the traditional automatic method were statistically significant.

The open-source tool OASIS [34] was used to perform the analysis. The results are included in the Supplementary Materials.

As shown in Table S2 (Supplementary Materials), very similar results were obtained for the manual and NN curves with a *p*-value of 0.7454.

When comparing the curve obtained with the traditional automatic method with the curve obtained with the new method (NN), a *p*-value of 0.6075 was obtained; therefore, the improvements were not statistically significant.

## 4. Discussion

This paper proposed a method to automate lifespan assays, which, by combining traditional computer vision techniques with a neural network-based classifier, enables the number of live and dead *C. elegans* to be counted, despite low-resolution images.

As mentioned above, detecting dead *C. elegans* is a complex problem, since, in the last days of life, they hardly move, and these movements are only small changes in head posture and/or tail movements. This is compounded by other difficulties such as dirt appearing on the plate, hindering detection, and the slight displacements occurring when the plates are placed in the acquisition system.

These difficulties mean that solving the problem using traditional techniques requires image alignment in addition to manual adjustment of numerous parameters, making the use of neural networks an attractive proposition.

By using our method based on neural networks, we avoid having to perform alignments and feature design. Despite the advantages of neural networks, they have the difficulty of requiring large datasets to train them. In this work, we addressed this difficulty by manually labelling a small dataset (108 plates) and applying data augmentation techniques.

To generate a considerable increase in data, a simulator was implemented to scale the initial training dataset (666 sequences) to a final dataset on the order of 23,000 sequences. The results obtained showed that training the model with only the simulated dataset led to an improvement in the hit ratio of 10.40% compared to the baseline of the model, trained with the original dataset available. Furthermore, it was shown that training the model with a mixed dataset of simulated and original data improved the hit ratio by a further 3.20%, reaching a 83.66% hit rate in the classification of dead *C. elegans* and a 98.56% hit rate for live *C. elegans*. Errors in this method were mostly due to noise problems. These errors included cases such as stains that merged with the worm body, stains that caused the worm body to appear split, worm images in the border area, and worm aggregations. Examples of such noise cases are presented in Figure S1 (Supplementary Materials).

Regarding the final error on the lifespan curves, the proposed method achieved small error rates (3.54% ± 1.30% per plate) with respect to the manual curve, demonstrating its feasibility and, moreover, with slightly better results than the traditional vision techniques used as a reference. When obtaining the lifespan curves, several problems were encountered. In the first days, worms could be lost due to occlusions in the plate walls and aggregations; in the last days, false positives could occur due to plate soiling. These problems were reduced by using an edge motion detection technique and a postprocessing algorithm.

## Figures and Tables

**Table 1:** Resnet18-LSTM architecture used. The output size shows the size of the feature maps and the details of the resnet layers show the filter size, number of feature maps, and number of block repetitions.

Layer Name	Output Size	Layer Details
conv1	[Batch_size × seq_length, 64, 112 × 112]	7 × 7, 64, stride 2
maxpool	[Batch_size × seq_length, 64, 56 × 56]	3 × 3, stride 2
layer1	[Batch_size × seq_length, 64, 56 × 56]	$\left[\begin{array}{c} 3\times 3,\;64\\ 3\times 3,\;64 \end{array}\right]\times 2$
layer2	[Batch_size × seq_length, 128, 28 × 28]	$\left[\begin{array}{c} 3\times 3,\;128\\ 3\times 3,\;128 \end{array}\right]\times 2$
layer3	[Batch_size × seq_length, 256, 14 × 14]	$\left[\begin{array}{c} 3\times 3,\;256\\ 3\times 3,\;256 \end{array}\right]\times 2$
layer4	[Batch_size × seq_length, 512, 7 × 7]	$\left[\begin{array}{c} 3\times 3,\;512\\ 3\times 3,\;512 \end{array}\right]\times 2$
av pool	[Batch_size × seq_length, 512, 1 × 1]	-
LSTM	[Batch_size, seq_length, 256]	In_features = 512 Hidden size = 256
linear	[Batch_size, 2]	In_features = 256 Out_features = 2
softmax	[Batch_size, 2]	-

**Table 2:** Number of sequences of each class (alive/dead) in the original dataset.

Alive	Dead
5696	847

**Table 3:** Number of synthetic sequences of each class (alive/dead) generated with the simulator.

Alive	Dead
11,220	11,220

**Table 4:** Confusion matrix for our model.

Label	Prediction Dead	Prediction Alive
dead	TD	FA
alive	FD	TA

**Table 5:** Results obtained by applying the initial detection algorithm to the original dataset.

Label	Prediction Stationary/Noisy	Prediction Alive
dead	833	14
alive	1072	4624

**Table 6:** Size of the original training and validation datasets.

Dataset	Sequences
Training	666
Validation	1000

**Table 7:** Mixed dataset.

Dataset	Live Worm Sequences	Dead Worm Sequences
Original	572	333
Simulated	11,220	11,220
Replicated	-	239
Total	11,792	11,792

**Table 8:** Comparison of model accuracy when trained with different datasets and validated with the original validation set.

Training Dataset	Training Size	Validation True Dead Rate	Validation True Live Rate	Mean	Improvement
Original	666	78.60%	65.40%	72.00%	-
Simulated	22,440	81.40%	83.40%	82.40%	10.40%
Mixed	23,584	86.00%	85.20%	85.60%	3.20%

**Table 9:** Confusion matrix of the validation sequences classified by the neural network with the model trained with a mixed dataset.

Label	Prediction Dead	Prediction Alive
Dead	430	70
Alive	74	426

**Table 10:** Total confusion matrix.

Label	Prediction Dead	Prediction Alive
Dead	430	84
Alive	74	5050

**Table 11:** Final results of the full classification method with the validation data.

True Dead Rate	True Live Rate	Mean
83.66%	98.56%	91.11%

## Data Availability

We created a repository on github: https://github.com/AntonioGarciaGarvi/C.-elegans-alive-dead-classification-using-deep-learning (accessed on 19 July 2021) with a demo to show some examples of how our model classifies a *C. elegans* as alive or dead using a sequence of three images corresponding to the current day, the day before, and the day after.

## Supplementary Materials

The following are available online at https://www.mdpi.com/article/10.3390/s21144943/s1, Table S1: Survival analysis, Table S2: Log-rank test obtained with the open source tool OASIS [34], Figure S1: Noise examples, Figure S2: N2 lifespan curve, Figure S3: *Daf-2* lifespan curve.
