# Supplementary material for: Towards Lifespan Automation for Caenorhabditis elegans Based on Deep Learning: Analysing Convolutional and Recurrent Neural Networks for Dead or Live Classification
Source: Sensors (Basel). 2021 Jul 20;21(14):4943. doi: 10.3390/s21144943 (PMC8309694; doi:10.3390/s21144943)
Supplement: Supplementary file 1 [file sensors-21-04943-s001.zip › sensors-1258645-supplementary.pdf]

Towards lifespan automation for *Caenorhabditis elegans* based on deep learning: analysing convolutional and recurrent neural networks for dead or live classification.

Antonio García Garví [1], Joan Carles Puchalt [1], Pablo E. Layana Castro [1], Francisco Navarro Moya[1] and Antonio-José Sánchez-Salmerón [1,*]

[1] Instituto de Automática e Informática Industrial, Universitat Politècnica de València, 46022 Valencia, Spain
*Correspondence: asanchez@isa.upv.es

Table of contents

1. **Statistical analysis** 3
2. **Noise examples** 4
3. **Shorter/longer lifespan validation** 5

1. Statistical analysis

Table S1. Survival analysis. This table shows the accuracy of the different methods in obtaining the mean/median lifespan.

Name	No. of subjects	Restricted mean			Age in days at % mortality			
		Days	Std. Error	95% C.I.	25%	50%	75%	95% Median C.I.
**Manual**	111	15.60	0.32	14.97 ~ 16.24	13	15	19	14.0 ~ 16.0
**Automatic**	111	14.96	0.33	14.31 ~ 15.62	12	14	19	13.0 ~ 14.0
**NN**	111	15.22	0.35	14.53 ~ 15.90	13	14	20	14.0 ~14.0

Table S2. Log-rank test obtained with the open source tool OASIS [34].

Condition	Statistics		
	Chi^2	P-value	Bonferroni P-value
**Manual *v.s.* Automatic**	0.77	0.3812	0.7623
**Manual *v.s.* NN**	0.11	0.7454	1.000
**Automatic *v.s.* Manual**	0.77	0.3812	0.7623
**Automatic *v.s.* NN**	0.26	0.6075	1.000
**NN *v.s.* Manual**	0.11	0.7454	1.000
**NN *v.s.* Automatic**	0.26	0.6075	1.000

2. Noise examples

Figure S1. Noise examples. This figure shows examples of image sequences in which noise appears, causing misclassification as dead or alive. Examples (a) and (b) show stains that partially merge with the worm's body. Examples (c) and (d) show stains that cause the worm's body to appear split. Examples (e) and (f) show wall zone errors. Examples (g) and (h) show cases of aggregation.

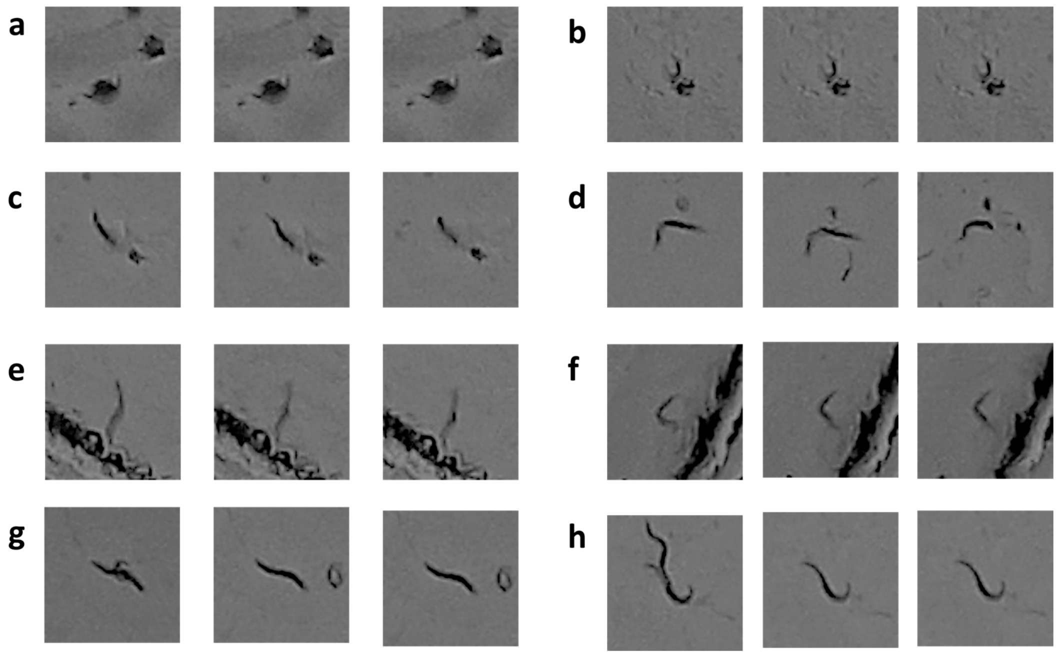

3. Shorter/longer lifespan validation

This section shows a comparison between curves obtained in assays with strain N2 and strain *daf*-2 (CB1370, *daf-2* (*e1370*)). Thus, it can be seen how the proposed method is able to classify adult *C. elegans* between short-lived and long-lived worms.

Note: It should be taken into account that part of the sequences of these assays have been used by the neural network in the training phase.

The following figure (Figure S2) shows the curve obtained in an assay with *C. elegans* strain N2 for a population n=100 distributed in 8 Petri dishes. For the post-processing of the curve, day 14, which is the mean-life of the strain, was used as the threshold.

Figure S2. N2 lifespan curve. Manual counting (blue curve), automatic counting traditional method (orange curve) and counting with the proposed method (grey curve) are compared. The horizontal axis shows the days of the experiment and the vertical axis shows the proportion of live *C. elegans.*

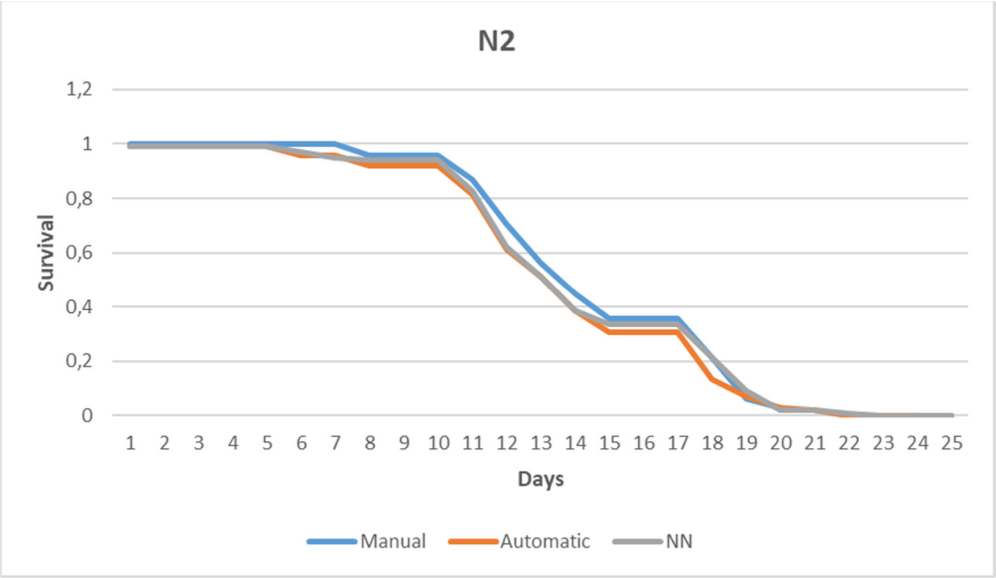

The curve obtained with the proposed method showed an average error in the analysis per plate of 2.73 ± 2.52% while the traditional algorithm obtained 4.42 ± 2.14%. In the analysis of the error per condition, an average error was obtained on each day of 2.55 ± 2.01% compared to 3.53 ± 2.57% for the traditional algorithm.

The curve obtained from an assay with *C. elegans* strain *daf-2* for a population n=100 distributed in 8 Petri dishes is shown below (Figure S3). For the post-processing of the curve, day 42, which is the mean-life of the strain, was used as the threshold.

Figure S3. *Daf-2* lifespan curve. Manual counting (blue curve), automatic counting traditional method (orange curve) and counting with the proposed method (grey curve) are compared. The horizontal axis shows the days of the experiment and the vertical axis shows the proportion of live *C. elegans*.

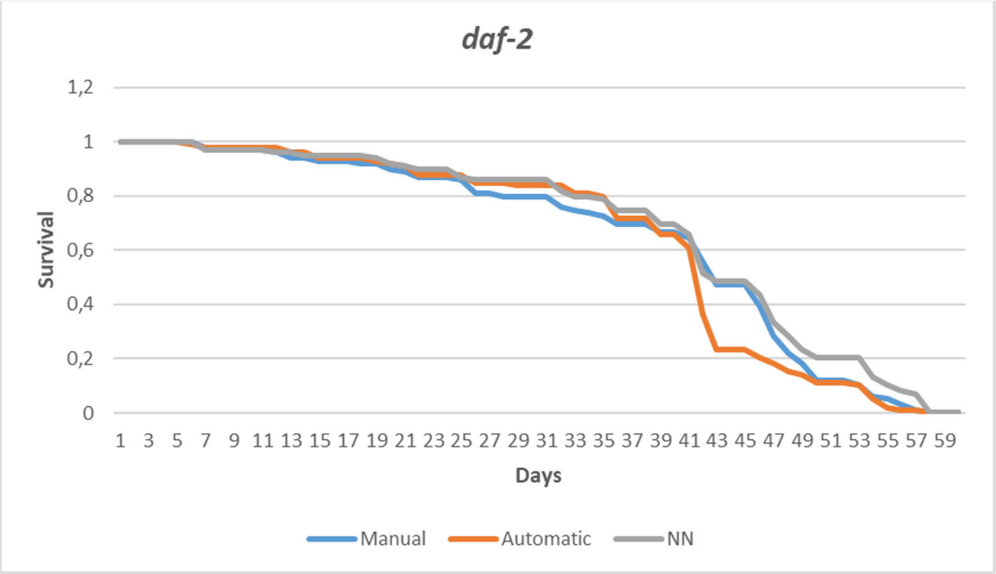

The curve obtained with the proposed method showed an average error in the analysis per plate of 4.10 ± 1.15% while the traditional algorithm obtained 8.41 ± 3.68%. In the analysis of the error per condition, an average error was obtained on each day of 3.40 ± 2.66% compared to 4.09 ± 6.14% for the traditional algorithm.
